# *De Novo* Assembly, Characterization and Functional Annotation of Pineapple Fruit Transcriptome through Massively Parallel Sequencing

**DOI:** 10.1371/journal.pone.0046937

**Published:** 2012-10-16

**Authors:** Wen Dee Ong, Lok-Yung Christopher Voo, Vijay Subbiah Kumar

**Affiliations:** Biotechnology Research Institute, Universiti Malaysia Sabah, Kota Kinabalu, Sabah, Malaysia; East Carolina University, United States of America

## Abstract

**Background:**

Pineapple (*Ananas comosus* var. comosus), is an important tropical non-climacteric fruit with high commercial potential. Understanding the mechanism and processes underlying fruit ripening would enable scientists to enhance the improvement of quality traits such as, flavor, texture, appearance and fruit sweetness. Although, the pineapple is an important fruit, there is insufficient transcriptomic or genomic information that is available in public databases. Application of high throughput transcriptome sequencing to profile the pineapple fruit transcripts is therefore needed.

**Methodology/Principal Findings:**

To facilitate this, we have performed transcriptome sequencing of ripe yellow pineapple fruit flesh using Illumina technology. About 4.7 millions Illumina paired-end reads were generated and assembled using the Velvet *de novo* assembler. The assembly produced 28,728 unique transcripts with a mean length of approximately 200 bp. Sequence similarity search against non-redundant NCBI database identified a total of 16,932 unique transcripts (58.93%) with significant hits. Out of these, 15,507 unique transcripts were assigned to gene ontology terms. Functional annotation against Kyoto Encyclopedia of Genes and Genomes pathway database identified 13,598 unique transcripts (47.33%) which were mapped to 126 pathways. The assembly revealed many transcripts that were previously unknown.

**Conclusions:**

The unique transcripts derived from this work have rapidly increased of the number of the pineapple fruit mRNA transcripts as it is now available in public databases. This information can be further utilized in gene expression, genomics and other functional genomics studies in pineapple.

## Introduction

The pineapple (*Ananas comosus* var. comosus), a member of the Bromeliaceae, is an economically important tropical fruit. Pineapple, along with three other dominant tropical fruits (mango, papaya and avocado), account for approximately 75% of global flesh tropical fruit production [1]. Despite its economic contribution, research on the genetics of the crop is scarce. This can be seen by the low number of pineapple genome sequences that are available in the public database. As of December 2011, the number of pineapple ESTs available in NCBI dbEST accounts to only about 6,000.

Pineapple is a non-climacteric fruit, as with grape, citrus, strawberry and pepper. In pineapple, there is no extra accumulation of starch during the ripening once the fruit is harvested or detached from the mother plant [2]. This is in contrast to climacteric fruits (such as tomato, papaya and banana), where a burst of respiration and a spike in ethylene causes the conversion of starch into sugar allowing the fruit to sweeten during fruit ripening. The unique ripening mechanism of non-climacteric fruit development is still unclear [3]–[4]. The underlying ripening pathways of the non-climacteric fruit can be deduced through the analysis of the transcripts being expressed.

Recent development in functional genomics has enabled the study of plant and organisms at the whole-transcriptomic level [5]. The emergence of the next generation sequencing (NGS) technology, allows for the rapid discovery of transcripts (including rare transcripts) and the quantification of gene expression. The applications of 454 (Roche), Solexa (Illumina) and/or Solid (Applied Biosystem) massively parallel sequencing, has been widely used to sequence any organism of choice at both the genomic or transcriptomic level. Utilization of short reads has been proven to effectively facilitate the *de novo* genome assembly [6], [7] and reference assembly of transcriptomic data [8]. It has also been shown that short reads have been effectively assembled and used in the discovery of genes and in the study of gene expression [9].

This paper reports on the millions of paired-end Solexa reads that were generated to reveal the transcriptomic profiles of the pineapple fruit. This dataset obtained through *de novo* assembly will be the first pineapple transcriptomic data generated from massively parallel sequencing. The information provides a good platform for further gene expression, genomics functional genomics and the non-climacteric ripening in pineapple.

## Results and Discussion

### Paired-end sequencing and *de novo* assembly

A total of about 4.7 (52.7%) million reads were obtained from mRNA-seq whole transcriptome sequencing of the pineapple fruit flesh. These were then assembled into 28,728 unique transcripts (UTs) using k-mer 47. The assembly produced 6.4 Mbp of reads with a N50 value of 223 bp. The length of the UTs generated from the *de novo* assembly ranged from 100 bp to 3.8 kb. The percentage of reads used in the generation of UTs obtained in this report was higher compared to those assembled (Solexa reads) in sweet potato root transcriptome [10]. On the other hand, in coral larval [11] and *Artemisia annua* [12] the percentage of reads used in the assembly were slightly higher compared to pineapple. However, this may be due to the different sequence length produced of the platforms i.e longer reads are produced from the 454/Roche compared to Illumina GA. In addition, the present of alternative splicing region in the transcripts also will hinder the assembly of long sequences [13]. Aside from this, the presence of repeats including both the simple and complex repeats was also likely factors contributing to be difficulty in assembly [14].

Estimating the number of genes and the level of transcripts coverage represented in an EST collection or assembly is important. To compute this, indirect evaluation of the pineapple transcriptome coverage was performed by comparing the number UTs generated from the assembly (28,728) and the mean length (n50 value = 223 bp) to the Arabidopsis thaliana genes.

Assuming a similar number of genes occur in pineapple as in A. thaliana, 25,000 genes with an average length of 2,000 bp [15]. This assembly was able to cover 0.13× transcripts of the pineapple transcriptome. The results was fairly good as the number of pineapple genes was solely evaluated from assembly of one fruit tissue and the UTs generated were assembled without a reference genome. Deep sequencing with various pineapple tissues and stages or reference assembly by using pineapple ESTs or nearest genome sequence will eventually fill up the gaps and give a more thorough transcripts representation in pineapple [16].

### Characterization by Similarity Search

In order to make an assessment for the putative identities of the assembly, all the UTs were subjected to BLASTx similarity search. A similarity search against the non-redundant (nr) NCBI database showed that 16,932 (59%) UTs had significant matches with e-values of ≤10−6 (Table 1). Of these, 13,407 matched to known genes while 3,525 matched to unknown genes. A large portion of the unknown genes were hypothetical proteins. The remaining 11,796 (41%) UTs resulted in no significant hits. The identification of un-characterized sequences from cDNA libraries ranges considerably with most being reported to be between 35–50% [17]. In the characterization of apple EST sequences, approximately 40% of the unique transcripts from the assembly did not exhibit significant similarity [18] while in strawberry ESTs, 43.9% were without significant similarity [19]. This was similar with pineapple, where 40% of the UTs were above the cut off value (Table 1). The high percentage of the pineapple transcripts in this study with no hits to the non-redundant (nr) protein database indicate that there is enormous potential for the discovery of new genes in this plant besides providing the possibility of new identification of gene networks and also the fruit ripening pathways. The portion of the UTs that resulted in ‘no hits’ to the nr database is mainly due to the short UTs length although obtaining 30–40% of sequences more than the cut-off value is quite common in many studies, especially in studies involving large-scale sequencing [11], [20]–[21].

**Table 1:** Summary of the assembly and annotation of pineapple UTs.

	Number of sequences (%)
**1. Assembly**	
Total number of reads	9,000,000
Total number of reads used for assembly	4,745,237
Total unigenes generated	28,728
**2. Similarity Search**	
Unigenes matching to known genes	13, 407 (46.7%)
Unigenes matching to unknown genes	3,525 (12.3%)
Unigenes matching to no hits	11,796 (41%)
**3. Functional Annotation**	
Unigenes without mapping	1,425
Unigenes with annotation	14,510
Number of GO terms assigned	65,637

**Figure 1:**
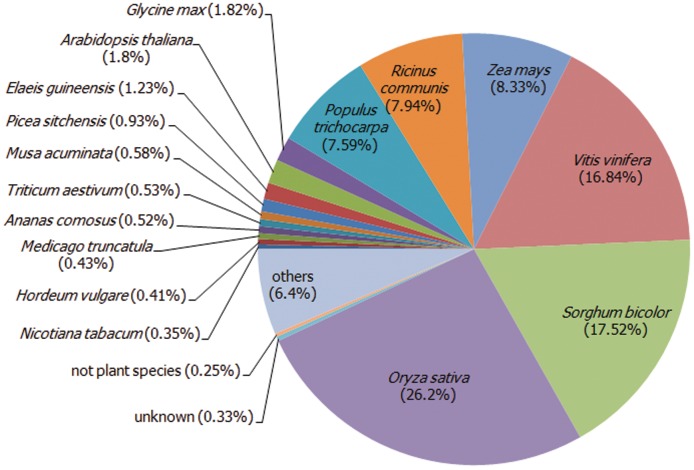
Species distribution of pineapple UTs from the velvet *de novo* assembly.

Of the UTs with matched similarities to the nr protein database, 452 (2.7%) had an e-value of e-100 or less and are considered highly significant. The moderately significant are those with e-values between e-20 to e-99. About 8,957 (52.9%) of the pineapple UTs were moderately similar to the return hits. Finally, the remaining 7,523 (44.4%) UTs were assigned weak homology i.e. e-values between e-7 to e-19. The overall e-value distribution pattern was similar with those characterized from oyster ESTs from oyster [21]. Organism distribution based on the BLASTx analysis of the UTs showed that the UTs hit a range of plant species. Among the various plants that have protein sequences in GenBank, the pineapple UTs had the highest number of hits to rice (*Oryza sativa*), by almost a quarter portion (26.2%). This was followed by *Sorghum bicolor* (17.52%), *Vitis vinifera* (16.84%), *Zea mays* (8.33%), *Ricinus communis* (7.94%) and *Populus trichocarpa* (7.59%). The hit to *Ananas comosus* was only 0.52% while, hits to non-plants organisms make up to a total of 0.25% (Figure 1).

Both the pineapple and rice are monocotyledons, which may explain for the high similarities that were observed. The high similarity of the pineapple UTs to the rice genes may provide for the possibility of using the rice's ESTs as a reference for future assembly using next generation sequencing. Collins and co-workers [22] showed that they managed to efficiently assemble the *Pachycladon enysii*, a polyploid plant by using a diploid plant as a reference genome. It is worth mentioning that the relatively small number of UTs matching to the pineapple protein sequences suggests that this assembly identified a considerable large group of genes previously unidentified in pineapple.

### Functional characterization by GO annotation

GO database, is a platform allowing a standardized gene functional annotation of the properties of genes using controlled vocabulary annotations which is categorised into the three main ontologies i.e. Molecular Function, Biological Processes and Cellular Component [23]. By using the Blast2GO program, the UTs were annotated against the non-redundant (nr) Genbank database. Next, the nr annotated UTs were then mapped against the GO database to retrieve the GO terms. In total 65,637 GO terms were assigned to all 15,507 mapped UTs with an average of one UT assigned to four GO terms. Of these, the majority of the GO terms were assigned to Molecular function (25,033, 38.1%) followed by Biological process (20,672, 31.5%) and the least were categorized under the Cellular component (19,932, 30.4%).

**Figure 2:**
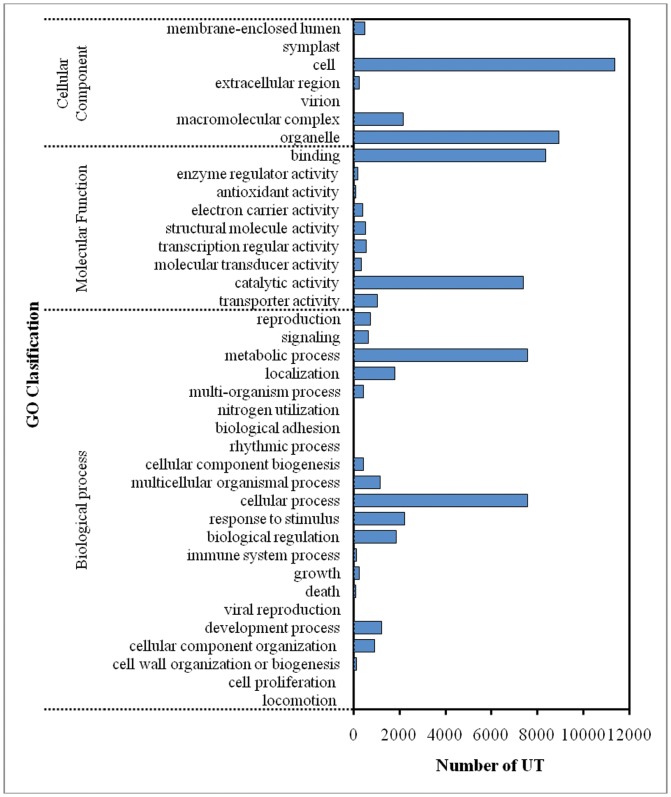
Gene Ontology classification of the pineapple UTs distribution in Cellular component, Molecular function and Biological process ontology.

Under the Cellular component ontology, proteins involved in cell and organelle development was dominant while in Molecular function ontology, proteins for binding and catalytic activity were generally encoded by the pineapple UTs. Biological process ontology distribution on the other hand, contains mainly proteins involved in cellular processes and metabolic processes (Figure 2). This pattern of distribution is typically seen in the transcriptome of samples undergoing development process [24]. During the development process of pineapple until it fully ripen on plant, biological processes involves cell development and cellular changes. Other proteins involved in the biological process ontology observed were those responsible for stress response, biological regulation, reproduction, developmental processes and multicellular organismal process. All these categories indicate that the pineapple fruit undergoes multiple processes of development and stresses.

### Functional classification by KEGG

In order to identify the active biological pathways in pineapple, the assembled UTs were used to obtain the Enzyme Commission (EC) when annotated against the KEGG database. A total of 5579 UTs were assigned to 7712 EC. The ECs were subsequently grouped into 126 biochemical pathways, of which 122 pathways were found to be involved in fruit metabolism. The presence of abundant metabolic pathways was also seen in the transcriptome of *Citrus sinensis* during fruit development [25]. Also, in the proteomic analysis of grape skin, the changes in the proteome among different stages of ripening mainly involve metabolic pathways [26]. This suggests that there are a high number of metabolic activities occurring during the fruit development of pineapple.

**Figure 3:**
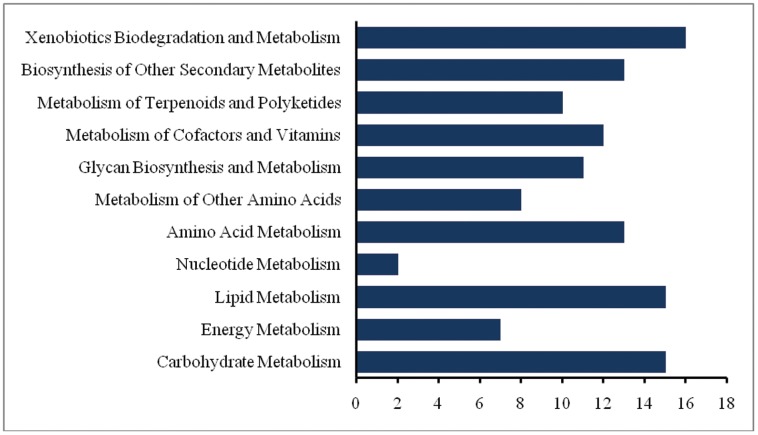
Classification of the sub-categories involved in the pineapple metabolism.

Enzymes involved in pineapple metabolism were further classified into 11 sub-categories. There are considerably higher portions of enzymes participate in the metabolism of lipid (15 pathways), carbohydrate (15 pathways), and xenobiotics biodegradation and metabolism (156 pathways) (Figure 3). This indicates that there are enormous cell walls activities, starch and sucrose synthesis and many other health-related compounds being synthesis during the ripe stage pineapple fruit. This is particularly similar with the analysis of Expressed Sequence Tags in apple [27] and kiwi fruit [28]. In transcriptome analysis of the mutant sweet orange, many genes were found to be involved in carbohydrates metabolism and were differentially expressed during fruit development [25]. The details of all pathways together with total UTs annotated to the pathways and the number of EC assigned to the pathways for of the sub-categories is provided in Additional file 1.

### Analysis of genes encoding important traits in pineapple using the assembled UTs

#### Fragrance Biosynthesis

**Figure 4:**
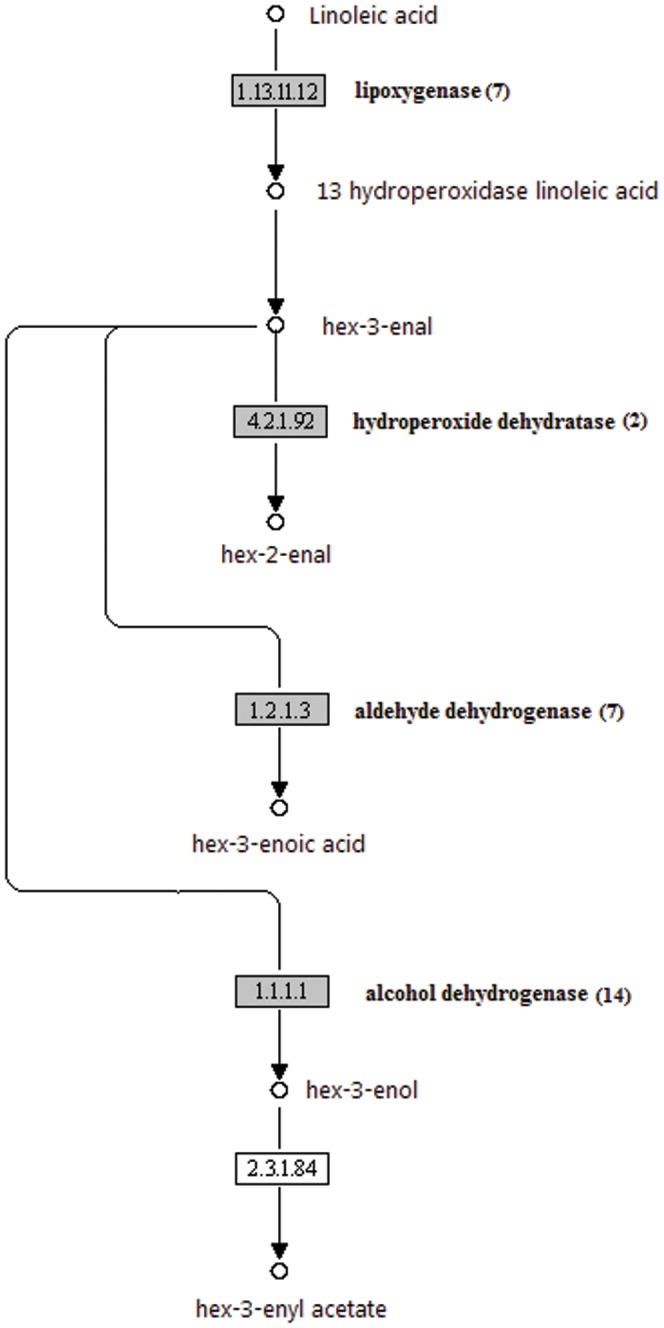
The terpene biosynthesis pathway. The pineapple transcripts encoding enzymes (highlighted) involved in the pathway was identified by BLASTx (e-06 cutoff). Numbers in bracket are the total number of UTs encode for the enzyme.

In the synthesis of fragrance in fruits, the volatiles compounds contributing aroma volatiles include the terpenes, alcohols, aldehydes, esters and aromatic acid [29]. Terpenoid and esteric compounds were predominantly found to contribute in the aroma biosynthesis of plants and fruits [30]. The terpene biosynthesis pathways synthesize compounds that include the monoterpenes, sesquiterpenes, diterpenes, triterpenes, carotenoids, ubiquinone and other terpenoid-quinones. The key enzyme that starts the terpene biosynthesis is the mevalonate kinase (Figure 4). The mevalonate kinase with the EC number of 2.7.1.36 were encoded by 4 UTs (EC:2.7.1.36; 4 UTs) react to convert mevalonate to 5-phosphomevalonate. Next, phosphomevalonate kinase (EC:2.7.4.2; 6 UTs) convert the compound to 5-diphosphomevalonate. Melavonate diphosphate decarboxylase (EC:4.1.1.33) which are responsible for the production of isopentenyl diphosphate were not detected in the annotated UTs.

However, isopentenyl diphosphate can be synthesized via the MEP/DOXP pathway where pyruvate is converted through a number of enzymatic steps. The enzymes involved and found to be encoded by the annotated UTs are 1-deoxy-D-xylulose-5-phosphate synthase (EC:2.2.1.7; 3 UTs), 1-deoxy-D-xylulose-5-phosphate reductoisomerase (EC:1.1.1.267; 3 UTs), 4-(cytidine 5′-diphospho)-2-C-methyl-D-erythritol kinase (EC:2.7.1.148; 3 UTs), 2-C-methyl-D-erythritol 2,4-cyclodiphosphate synthase (EC:4.6.1.12; 2 UTs), 4-hydroxy-3-methylbut-2-enyl diphosphate reductase (EC:1.17.1.2; 4 UTs) and isopentenyl-diphosphate Delta-isomerase (EC:5.3.3.2; 4 UTs) (Figure 4).

Next, conversion of isopentenyl diphosphate or/and dimethylallyl diphosphate to geranyl diphosphate (geranyl-PP) was carried out by dimethylallyltranstransferase (EC:2.5.1.1; 1 UT) for the synthesis of monoterpenes. The enzyme, Polyisoprene synthase, which is responsible for both the farnesyl diphosphate (farnesyl-PP) and geranyl geranyl diphosphate (geranyl geranyl-PP) synthesis of sesquiterpenes and diterpenes respectively, was not identified in the pineapple fruit assembly. However, the enzymes for triterpenes and carotenoids synthesis were found to be present in the fruit transcriptome. Two sequences encoding enzyme squalene monooxygenase (EC: 1.14.99.7; 2 UTs), which is involved in the conversion of squalene to sterol for the synthesis of triterpenes were identified. Another two sequences encoding phytoene synthase (EC:2.5.1.32; 2 UTs) which is involved in carotenoid synthesis was also discovered.

**Figure 5:**
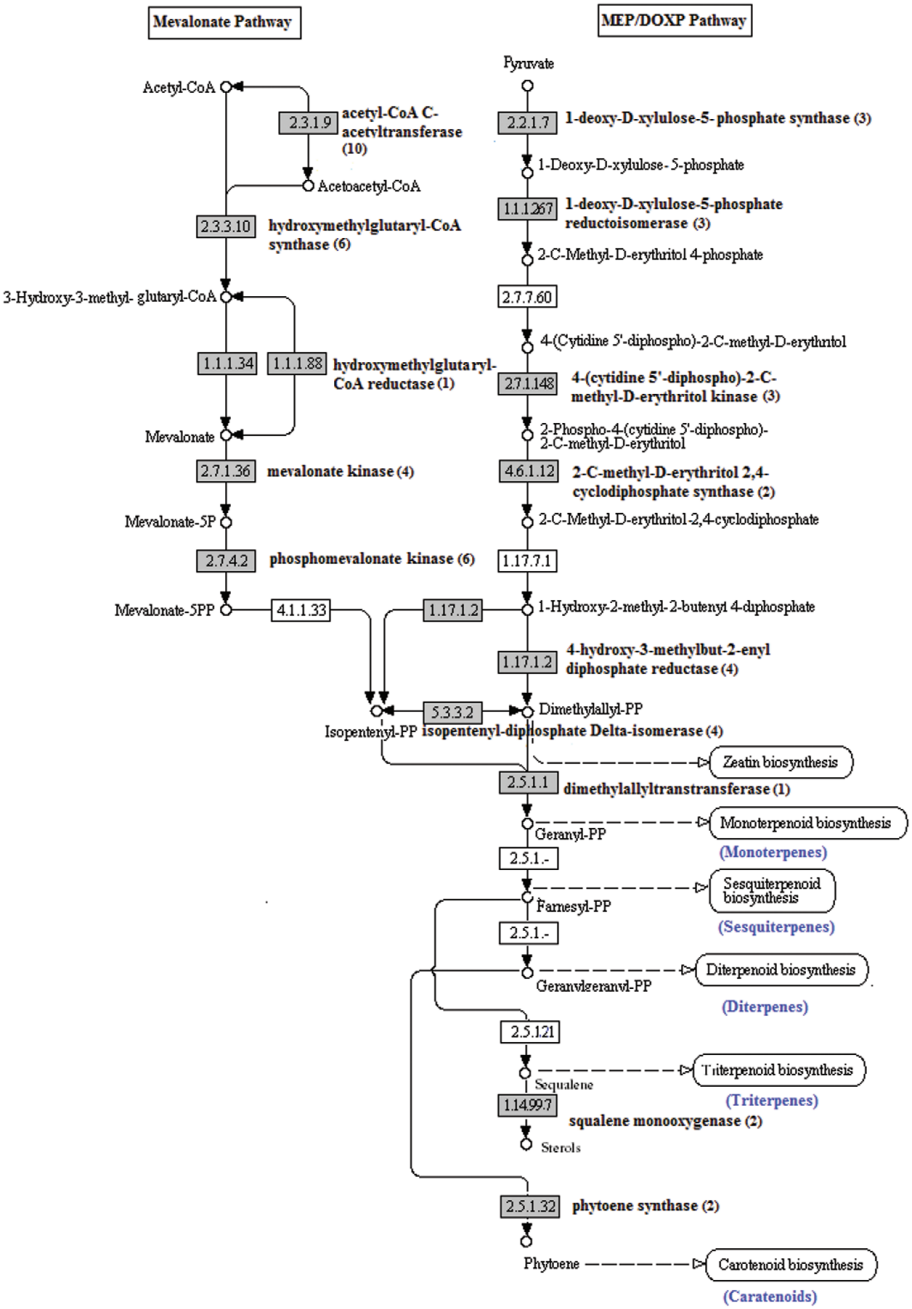
The straight chain ester biosynthesis from fatty acids. The pineapple transcripts encoding enzymes (highlighted) involved in the pathway was identified by BLASTx (e-06 cutoff). Numbers in bracket are the total number of UTs encode for the enzyme.

Ester compounds have been reported to be commonly synthesized in fruits,such as *Actinidia* [28], *Malus domestica* [27] and pineapple [31]. Four out of five enzyme involved in the straight chain ester biosynthesis were identified in pineapple. The first step in the synthesis involves the activity of the lipoxygenase enzyme (EC: 1.13.11.12; 7 UTs). It converts linoleic acid to 13 hydroperoxidase linoleic acid. From 13 hydroperoxidase linoleic acid, hydroperoxide lyase enzyme converts the compound to hex3-enal, which is then converted to hex-2-enal by hydroperoxide dehydratase (EC: 4.2.1.92; 2 UTs). The aldehydes, can either be converted to its acids by aldehyde dehydrogenases (EC: 1.2.1.3; 7 UTs) or to alcohols by alcohol dehydrogenese (EC: 1.1.1.1; 14 UTs). The alcohol, hex-3-enol, is later converted to ester by alcohol acyl transferases (Figure 5).

**Figure 6:**
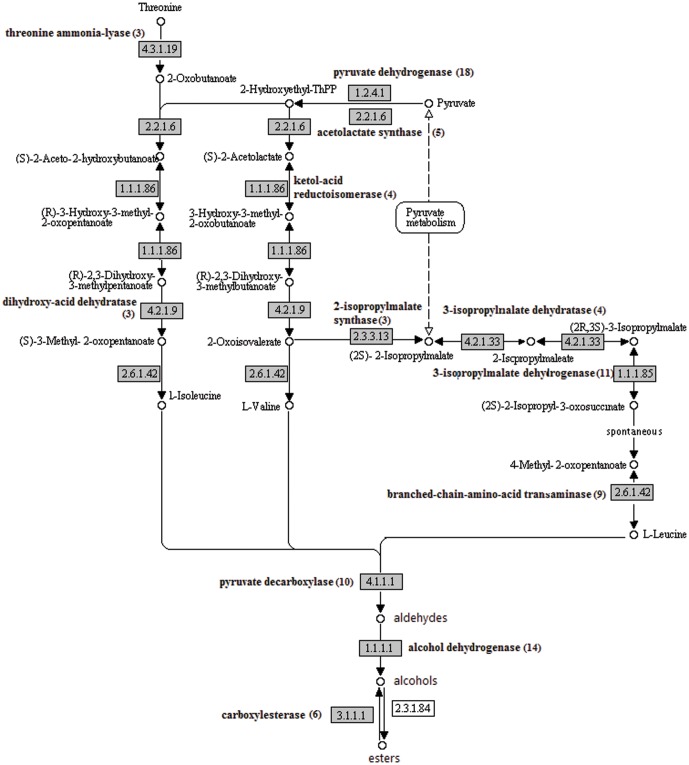
The branched chain ester biosynthesis. The pineapple transcripts encoding enzymes (highlighted) involved in the pathway was identified by BLASTx (e-06 cutoff). Numbers in brackets are the total number of UTs encode for the enzyme.

Branched chain esters are synthesized via isoleucine, valine and leucine [27]. The chain of enzymes involved in converting threonine and/or pyruvate to valine, leucine and isoleucine were identified in the pineapple UTs (Figure 6). They were threonine ammonia-lyase (EC: 4.3.1.19; 3 UTs), acetolactate synthase (EC: 2.2.1.6; 5 UTs), ketol-acid reductoisomerase (EC: 1.1.1.86; 4 UTs), dihydroxy-acid dehydratase (EC: 4.2.1.9; 3 UTs), branched-chain-amino-acid transaminase (EC: 2.6.1.42; 9 UTs), 2-isopropylmalate synthase (EC: 2.3.3.13; 3 UTs), 3-isopropylmalate dehydratase (EC: 4.2.1.33; 4 UTs), 3-isopropylmalate dehydrogenase (EC: 1.1.1.85; 11 UTs) and branched-chain-amino-acid transaminase (EC: 2.6.1.42; 9 UTs). The three acid syntheses are then converted to its aldehydes and alcohols by decarboxylase enzyme (EC: 4.1.1.1; 10 UTs) and alcohol dehydrogenase (EC: 1.1.1.1; 14 UTs), respectively. Alcohol acyl transferase will then convert the alcohols to ester.

#### Flavor Biosynthesis

**Figure 7:**
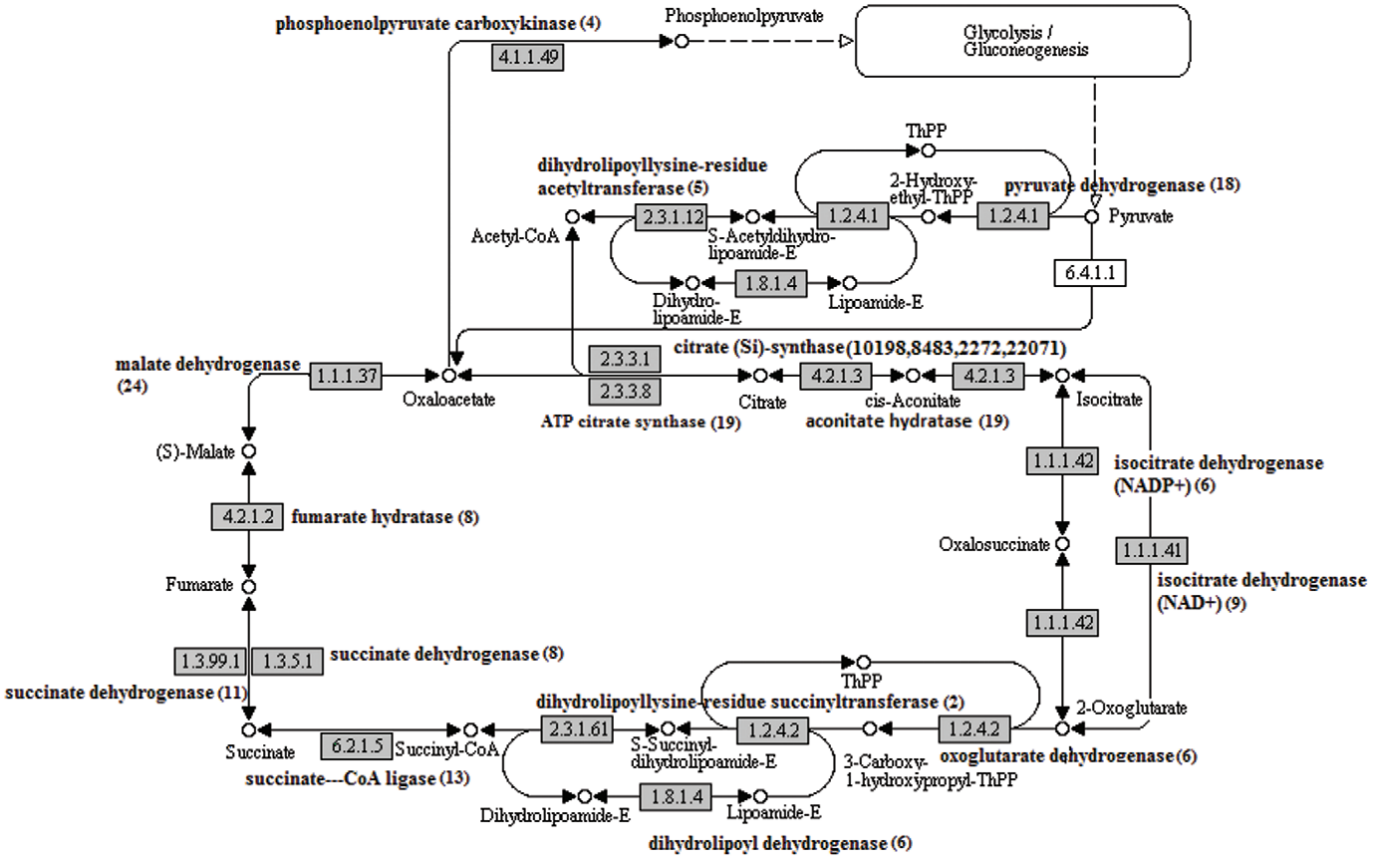
The citrate acid cycle. The pineapple transcripts encoding enzymes (highlighted) involved in the pathway was identified by BLASTx (e-06 cutoff). Numbers in brackets are the total number of UTs encode for the enzyme.

Flavor and aroma are closely related traits which give the best eating quality of the pineapple. Fruit acidity measured by the synthesis of the organic acid and the sweetness (sucrose metabolism) are the major factors of concern in the biosynthesis of flavor compounds. The organic acid synthesis (malic acid and citric acid) accumulations in the fruit involves the citrate acid cycle (Figure 7). The enzymes, citrate synthase (EC:2.3.3.1; 4 UTs), aconitate hydratase (EC:4.2.1.3; 20 UTs), phosphoenolpyruvate carboxylase (PEPC) (EC:4.1.1.31; 21 UTs), malate dehydrogenase (MDH) (EC:1.1.1.37; 24 UTs) and malic enzyme (EC:1.1.1.40; 5 UTs) were found to be encoded by the assembled UTs. Three of these enzymes, citrate synthase, aconitate hydratase and malate dehydrogenase were found be involved in the citrate acid cycle.

**Figure 8:**
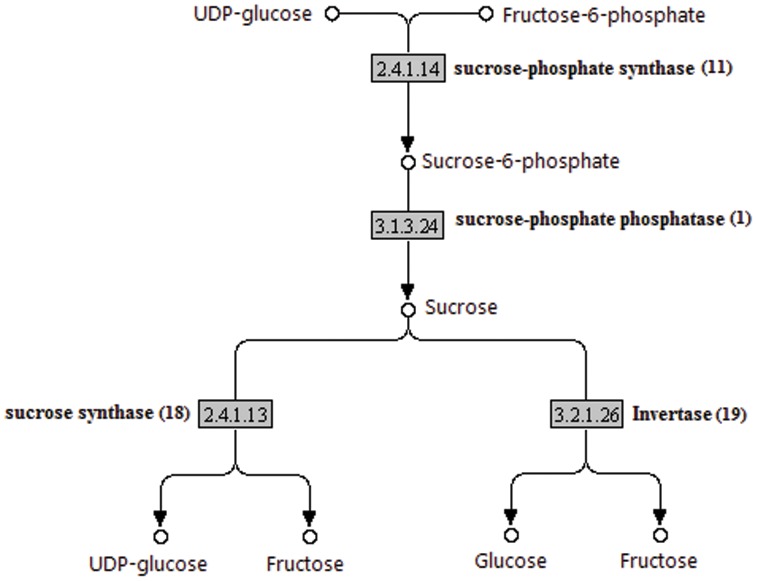
The sucrose metabolism. The pineapple transcripts encoding enzymes (highlighted) involved in the pathway was identified by BLASTx (e-06 cut off). Numbers in brackets are the total number of UTs encode for the enzyme.

A great number of enzymes involved in the starch and sucrose metabolism have been identified encoded by the pineapple UTs. The sucrose metabolism route involves four main enzymes. Sucrose phosphate synthase (SPS) (EC:2.4.1.14; 11 UTs) synthesizes sucrose-6-phosphate from UDP-glucose and fructose-6-phosphate. Next, sucrose-6-phosphate undergo hydrolysis to form sucrose by the action of sucrose-phosphate phosphatase (EC:3.1.3.24; 1 UT). The sucrose are then cleaved to sugar nucleotides either by sucrose synthase (EC:2.4.1.13; 18 UTs) and/or invertase (EC:3.2.1.26; 19 UTs) (Figure 8).

#### Texture/Structural Biosynthesis

**Figure 9:**
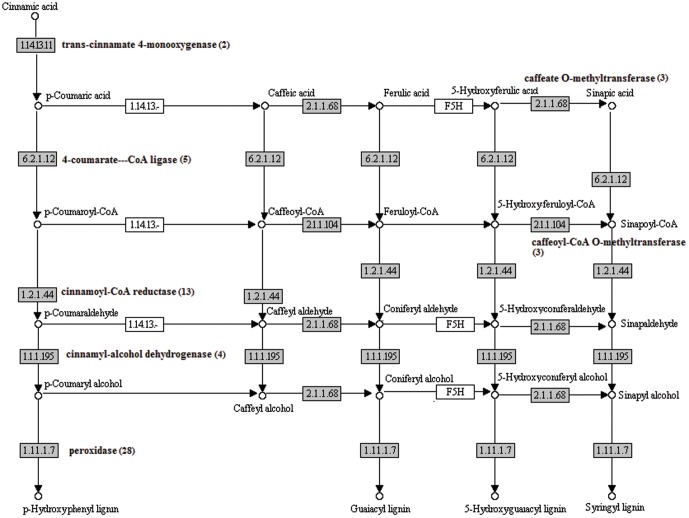
The lignin biosynthesis. The pineapple transcripts encoding enzymes (highlighted) involved in the pathway was identified by BLASTx (e-06 cutoff). Numbers in brackets are the total number of UTs encode for the enzyme.

Lignin is a type of polymer derived from the combination of aromatic monomers subunits [32]. Lignin was the second most abundant molecule after cellulose that provides plant with structural rigidity. Aside from the structural function, lignin also acts as an important component of fiber in the fruit and is beneficial for human health [33]. The initial step in its biosynthesis involves the deamination of phenylalanine to cinnamic acid. Phenylalanine are then converted to p-coumaric acid by trans-cinnamate 4-monooxygenase (EC:1.14.13.11; 2 UTs). p-coumaric acid are then converted to caffeic acid by coumarate 3-hydroxylase. From caffeic acid, caffeate O-methyltransferase (C-OMT) (EC: 2.1.1.68; 3 UT) then converts this compound to ferulic acid. Conversion from ferulic acid to 5-hydroxyferulic acid and then to sinapic acid was carried out by ferulate 5-hydroxlase (FSH) and C-OMT, respectively (Figure 9).

All the acid synthesized as mentioned above are then ligated with CoA to form it respective CoA acid derivatives by 4-coumarate-CoA ligase (EC:6.2.1.12; 5 UTs). These acids are then reduced to its corresponding aldehydes by cinnamoyl-CoA reductase (EC:1.2.1.44; 13 UTs). Next, the aldehydes are then reduced to its alcoholic compound by cinnamyl alcohol dehydrogenase (CAD) (EC: 1.1.1.195; 4 UTs). The final step of the lignin biosynthesis is the polymerization the monolignols by peroxidase (EC:1.11.1.7; 28 UTs). Other intermediate compound conversion enzyme found in pineapple was caffeoyl-CoA O-methyltransferase (EC: 2.1.1.104; 3 UTs) which convert coffeoyl-CoA to feruloyl-CoA and 5-hydroxy-feruloyl-CoA to sinapoyl-CoA.

#### Health-Related Compound Biosynthesis

The general vitamins that were found in many types of vegetables and fruits include thiamin (vitamin B1), riboflavin (vitamin B2), nicotinamide (vitamin B3), biotin (vitamin B7), folate (vitamin B9) and ascorbate (vitamin C) [34]–[35]. The pineapple UTs encoded some of the enzymes in the synthesis of the above health-related compounds. Only a small portion of the enzymes involved in the synthesis of thiamin, nicotinamide, biotin and ascorbate was identified. A more complete enzymatic activity was observed in the synthesis of riboflavin and folate. Many researchers have focus to increase folate in staple fruit as the intake is essential in normal diet [36]. Despite its importance, human and animals are not able to synthesis folate. This drives the identification of high folate content food and the biofortication of folate (enhancement of folate in plants). Biofortication of folate in tomato was has been conducted by crossing of transgenic tomato with PABA- (p-aminobenzoate) and pteridine-overproduction traits which managed to increase the folate content to more than 25-folds [37]. The same method may possibly be applied in pineapple, whereby the increase in the nutritional content of pineapple fruit may eventually increase its economic value.

## Materials and Methods

### Plant Material and mRNA Sequencing

Pineapple was collected from an experimental plot at a pineapple farm managed by a local farmer located at Kampung Babagon, Sabah. The pineapple sample was kindly provided and permitted by the farm owner. The sample was excised, packed and stored at −80°C. The total RNA of the ripe pineapple fruit tissue was extracted using method described by [38] with minor modifications. The RNA isolated was checked for yield (≥500 ng/µl) and quality by measuring both the A260/280 and A260/230 ratios (with a ratio of more than 1.6) using a NanoVue spectophotometer (GE Healthcare, USA). Total RNA volume of 30 µl (with the required yield and purity) was sent on May 2009 for paired-end mRNA sequencing with Genome Analyzer IIx, using the service provided by Illumina FastTrack, USA. A 75 bp paired-end sequencing protocol with insert sizes of 200 bp was employed.

### *De novo* Assembly by Velvet Software

Velvet *de novo* software was used to assemble the 75 bp paired-end reads. The software algorithm involved the construction of de Bruijn graph for the sequence assembly [39]. Therefore, different k-mers (or hash number) were initially used to assess the best assembly of the paired-end reads. It was found that the assembly with k-mer of 47 provided the best output for the transcriptomic assembly. The unique transcripts (UTs) obtained from assembly using k-mers of 47 were then used for subsequent downstream analysis. The assembled UTs with sequence length longer than 200 bp have been deposited in the Transcriptome Shotgun Assembly (TSA) at NCBI under the accession number: JI400295–JI412109.

### Functional Characterization and Gene Ontology (GO) Annotation

All the UTs were then used for blast search against GenBank's non-redundant database. Blast matches were considered significant with E-value scores ≤10−6 using the BLASTx tool (http://blast.ncbi.nlm.nih.gov/Blast.cgi) [40]. Gene Ontology (GO) annotations were performed to retrieve molecular function, biological process and cellular component terms using Blast2GO (http://www.blast2go.org/) [23]. The sequences were loaded into the Blast2GO program, and BLASTx with a minimum E-value of 10−6 was performed by the program prior to mapping. The mapping step allowed the annotation of the sequences to the GO database for GO terms.

### Functional Classification by KEGG

Annotation against the Kyoto Encyclopedia of Genes and Genomes (KEGG) database (http://www.genome.jp/kegg/) were performed to obtain the link of enzymes integrating together genomic, chemical and network information [41] using the Blast2GO program. All the mapped sequences were annotated to the KEGG database to obtain the enzyme commission (EC) number. The EC were then mapped to the KEGG Pathway to obtain the KEGG Pathway-Maps.

## Supporting Information

Table S1 List of metabolism pathways in ripe pineapple fruit UTs mapped against KEGG database.
(DOC)
