# Supplementary material for: De Novo Assembly, Characterization and Functional Annotation of Pineapple Fruit Transcriptome through Massively Parallel Sequencing
Source: PLoS One. 2012 Oct 16;7(10):e46937. doi: 10.1371/journal.pone.0046937 (PMC3473051; doi:10.1371/journal.pone.0046937)
Supplement: Table S1 — List of metabolism pathways in ripe pineapple fruit UTs mapped against KEGG database. (DOC) [file pone.0046937.s001.doc]

**Table S1 - List of metabolism pathways in ripe pineapple fruit UTs mapped agains**t KEGG database.

| **Pathways** | **Total UTs** | **EC Distribution** |
| --- | --- | --- |
| **1. Carbohydrate Metabolism** |  |  |
| Starch and sucrose metabolism | 300 | 37 |
| Glycolysis / Gluconeogenesis | 235 | 24 |
| Pyruvate metabolism | 193 | 20 |
| Galactose metabolism | 142 | 16 |
| Citrate cycle (TCA cycle) | 141 | 16 |
| Amino sugar and nucleotide sugar metabolism | 133 | 30 |
| Propanoate metabolism | 127 | 12 |
| Pentose phosphate pathway | 119 | 14 |
| Butanoate metabolism | 98 | 16 |
| Glyoxylate and dicarboxylate metabolism | 84 | 13 |
| Inositol phosphate metabolism | 81 | 15 |
| Fructose and mannose metabolism | 78 | 15 |
| Ascorbate and aldarate metabolism | 55 | 10 |
| Pentose and glucuronate interconversions | 42 | 10 |
| C5-Branched dibasic acid metabolism | 18 | 2 |
| **2. Energy Metabolism** |  |  |
| Oxidative phosphorylation | 165 | 9 |
| Carbon fixation in photosynthetic organisms | 156 | 17 |
| Reductive carboxylate cycle (CO2 fixation) | 126 | 9 |
| Methane metabolism | 70 | 7 |
| Photosynthesis | 47 | 2 |
| Nitrogen metabolism | 46 | 17 |
| Sulfur metabolism | 42 | 12 |
| **3. Lipid Metabolism** |  |  |
| Glycerophospholipid metabolism | 96 | 16 |
| Fatty acid metabolism | 95 | 11 |
| Glycerolipid metabolism | 81 | 14 |
| alpha-Linolenic acid metabolism | 72 | 8 |
| Biosynthesis of unsaturated fatty acids | 54 | 6 |
| Fatty acid biosynthesis | 41 | 10 |
| Sphingolipid metabolism | 39 | 7 |
| Fatty acid elongation in mitochondria | 32 | 5 |
| Ether lipid metabolism | 28 | 2 |
| Steroid biosynthesis | 26 | 10 |
| Synthesis and degradation of ketone bodies | 21 | 4 |
| Primary bile acid biosynthesis | 16 | 1 |
| Steroid hormone biosynthesis | 15 | 4 |
| Arachidonic acid metabolism | 15 | 4 |
| Linoleic acid metabolism | 13 | 2 |
| **4. Nucleotide Metabolism** |  |  |
| Purine metabolism | 272 | 35 |
| Pyrimidine metabolism | 99 | 20 |
| **5. Amino Acid Metabolism** |  |  |
| Valine, leucine and isoleucine degradation | 124 | 19 |
| Tryptophan metabolism | 104 | 12 |
| Cysteine and methionine metabolism | 99 | 24 |
| Arginine and proline metabolism | 97 | 26 |
| Glycine, serine and threonine metabolism | 96 | 20 |
| Valine, leucine and isoleucine biosynthesis | 79 | 12 |
| Phenylalanine metabolism | 73 | 11 |
| Lysine degradation | 69 | 8 |
| Alanine, aspartate and glutamate metabolism | 68 | 20 |
| Tyrosine metabolism | 68 | 15 |
| Phenylalanine, tyrosine and tryptophan biosynthesis | 63 | 15 |
| Histidine metabolism | 37 | 11 |
| Lysine biosynthesis | 27 | 9 |
| **6. Metabolism of Other Amino Acids** |  |  |
| Glutathione metabolism | 89 | 16 |
| beta-Alanine metabolism | 77 | 10 |
| Selenoamino acid metabolism | 47 | 12 |
| Cyanoamino acid metabolism | 38 | 8 |
| Phosphonate and phosphinate metabolism | 10 | 2 |
| D-Glutamine and D-glutamate metabolism | 7 | 2 |
| Taurine and hypotaurine metabolism | 5 | 2 |
| D-Alanine metabolism | 3 | 1 |
| **7. Glycan Biosynthesis and Metabolism** |  |  |
| Other glycan degradation | 43 | 5 |
| N-Glycan biosynthesis | 40 | 8 |
| Glycosaminoglycan degradation | 24 | 2 |
| Glycosphingolipid biosynthesis - ganglio series | 24 | 2 |
| Glycosphingolipid biosynthesis - globo series | 20 | 4 |
| High-mannose type N-glycan biosynthesis | 8 | 1 |
| Glycosphingolipid biosynthesis - lacto and neolacto series | 7 | 2 |
| Glycosaminoglycan biosynthesis - heparan sulfate | 7 | 1 |
| Lipopolysaccharide biosynthesis | 4 | 2 |
| Peptidoglycan biosynthesis | 3 | 1 |
| O-Mannosyl glycan biosynthesis | 1 | 1 |
| **8. Metabolism of Cofactors and Vitamins** |  |  |
| Thiamine metabolism | 89 | 6 |
| Porphyrin and chlorophyll metabolism | 63 | 15 |
| Riboflavin metabolism | 48 | 9 |
| One carbon pool by folate | 40 | 13 |
| Pantothenate and CoA biosynthesis | 37 | 10 |
| Nicotinate and nicotinamide metabolism | 36 | 7 |
| Retinol metabolism | 17 | 2 |
| Vitamin B6 metabolism | 13 | 3 |
| Ubiquinone and other terpenoid-quinone biosynthesis | 12 | 3 |
| Folate biosynthesis | 7 | 4 |
